# Endophytic Fungus UJ3-2 from *Urtica fissa*: Antibacterial Activity and Mechanism of Action against *Staphylococcus aureus*

**DOI:** 10.3390/molecules29204850

**Published:** 2024-10-13

**Authors:** Fei Liao, Jie He, Renjun Li, Yanchun Hu

**Affiliations:** 1 Guizhou Vocational College of Agriculture, Qingzhen 551400, China; liaofeiyh@163.com (F.L.); 11317035@zju.edu.cn (R.L.); 2 Key Laboratory of Animal Disease and Human Health of Sichuan Province, College of Veterinary Medicine, Sichuan Agricultural University, Chengdu 611130, China

**Keywords:** endophytic fungi, *Staphylococcus aureus*, secondary metabolite, antimicrobial activity, biofilms

## Abstract

Taking the endophytic fungus UJ3-2, isolated from *Urtica fissa*, as the experimental material, this study aimed to explore the composition of its metabolites and the underlying mechanisms by which it inhibits *Staphylococcus aureus*. Initially, the MIC, MBC, inhibitory curves, biofilm growth, and extracellular nucleic acids and proteins of S. aureus in response to the metabolites were measured. Secondly, PI staining and SEM were used to evaluate the impact of the metabolites on the integrity of the cell wall and overall morphology of *S. aureus*. Additionally, UPLC-MS was employed to analyze the composition of the secondary metabolites. The UJ3-2 strain was identified as *Xylaria grammica* based on ITS sequencing and designated as *Xylaria grammica* UJ3-2. Our results revealed that the metabolites of UJ3-2 exhibited excellent in vitro antibacterial activity against *S. aureus*, with both MIC and MBC values of 3.125 mg/mL. The inhibitory curve confirmed that 1 MIC of UJ3-2 metabolites could completely inhibit the growth of *S. aureus* within 24 h. With increasing concentrations of UJ3-2 metabolites, the growth of *S. aureus* biofilms was significantly suppressed, and obvious leakage of nucleic acids and proteins was observed. PI fluorescence staining indicated that various concentrations of UJ3-2 metabolites disrupted the integrity of the *S. aureus* cell membrane. SEM observation revealed that the treated *S. aureus* surfaces became rough, and the bacteria shrank and adhered to each other, showing a dose-dependent effect. UPLC-MS analysis suggested that the main components of the fermented metabolites were 6-oxocineole (17.92%), (S)-2-acetolactate (9.91%), 3-methyl-cis,cis-muconate (4.36%), and 8-oxogeranial (3.17%). This study demonstrates that the endophytic fungus UJ3-2 exhibits remarkable in vitro antibacterial effects against *S. aureus*, primarily by enhancing the permeability of the *S. aureus* cell membrane, causing the leakage of its intracellular contents, and altering the bacterial surface morphology to inhibit the pathogen. The endophytic fungus UJ3-2 has a good antibacterial effect on *S. aureus*, which gives it certain application prospects in the screening and industrial production of new and efficient natural antibacterial active substances.

## 1. Introduction

*Staphylococcus aureus* (*S. aureus*) continues to be one of the most involved bacteria in animal and human diseases. This bacterium is found in the normal skin microbiota of both animals and humans, with a carriage rate of between 20 and 30% in the healthy human population [1]. Antibiotic resistance develops quickly in *S. aureus*, and the rise in multidrug-resistant forms is a major problem [2]. It has been reported that the annual mortality toll from antibiotic-resistant diseases has surpassed 10 million and that by 2050, it will outnumber cancer deaths [3]. Methicillin-resistant *S. aureus* (MRSA) represents a prevalent cause of multidrug-resistant infections, resulting in considerable morbidity and mortality [4]. The clinical manifestations and predisposing factors for infection differ among HA-MRSA, CA-MRSA, and LA-MRSA strains [5]. The morbidity and mortality consequences reinforce the need to urgently discover new effective solutions due to the inefficiency of traditional antibiotics. Therefore, alternative treatments represent a promising field of investigation due to the lack of new antibiotic classes.

Endophytes refer to bacteria or fungi that inhabit plant organs, are able to colonize within plant tissues, and do not cause significant harm to the host [6]. Plant–endophyte associations represent an inexhaustible source of novel metabolites, exhibiting significance from environmental, agricultural, and pharmaceutical perspectives [7,8]. Endophytic fungi are an important reservoir of therapeutically active compounds [9]. Hydrolytic enzymes from Amazonian endophytic fungi *Myrcia guianensis* effectively eradicate *S. aureus* biofilms, with combinations of lipase and protease showing total degradation, highlighting their potential as a tool against microbial biofilms [10]. *Penicillium brevicompactum* ANT13 from *Abies numidica* is a promising source of novel antimicrobials, effectively inhibiting multidrug-resistant *S. aureus* and dermatophytes, with ethyl acetate extract exhibiting significant activity [11]. A new nucleoside, cyclohexenone, and benzofuranone derivatives from *Aspergillus polyporicola* R2, with compounds 11-dehydrocurvularin, 11α-hydroxycurvularin, and 2,5-dimethyl-3(2H)-benzofuranone, exhibit antimicrobial activity against MRSA, *Salmonella*, and *Fusarium graminearum*, respectively [12].

Faced with the increasingly severe challenge of antibiotic resistance, this global issue has posed a significant threat to human health that cannot be ignored. Therefore, actively exploring and deeply mining the resources of endophytic fungi in plants has become one of the most important ways for us to find and develop new and efficient antibacterial drugs [13,14]. Based on the previously screened endophytic fungus UJ3-2 from *Urtica fissa,* with antibacterial activity against *S. aureus*, *Escherichia coli*, and *Salmonella*, using tetracycline as a positive drug, it was found that UJ3-2 exhibited the best antibacterial effect against *S. aureus*, but its antibacterial components and mechanisms need further research. Therefore, this experiment aims to explore the antibacterial components and their mechanisms of action in the metabolites of the endophytic fungus UJ3-2 by determining its antibacterial activity against *S. aureus* and its effects on cell wall and membrane integrity, biofilm, and bacterial structure. It is expected to provide practical evidence for the study of the antibacterial mechanism of pathogens and a theoretical basis for screening novel and natural antibacterial active substances.

## 2. Result

### 2.1. Identification of Endophytic Fungus UJ3-2

**Figure 1:**
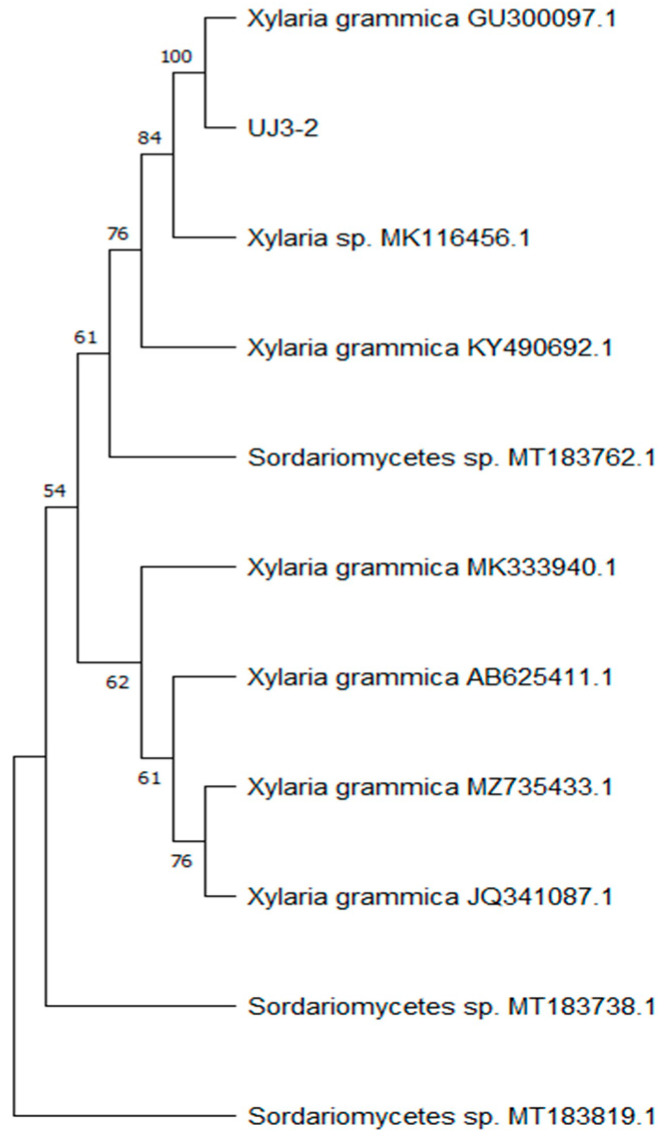
Phylogenetic tree based on ITS rDNA sequence.

After extracting the fungal genomic DNA, the ITS sequence was amplified, and the amplified PCR products were sent for sequencing. A phylogenetic tree of endophytic fungi was constructed, as shown in Figure 1, which indicated 100% homology with *Xylaria grammica*, thus identifying it as *Xylaria grammica*. Meanwhile, the sequencing data were uploaded to the NCBI database under sequence number PP957465.1.

### 2.2. Metabolome Analysis of UJ3-2 Fermentation Products

**Figure 2:**
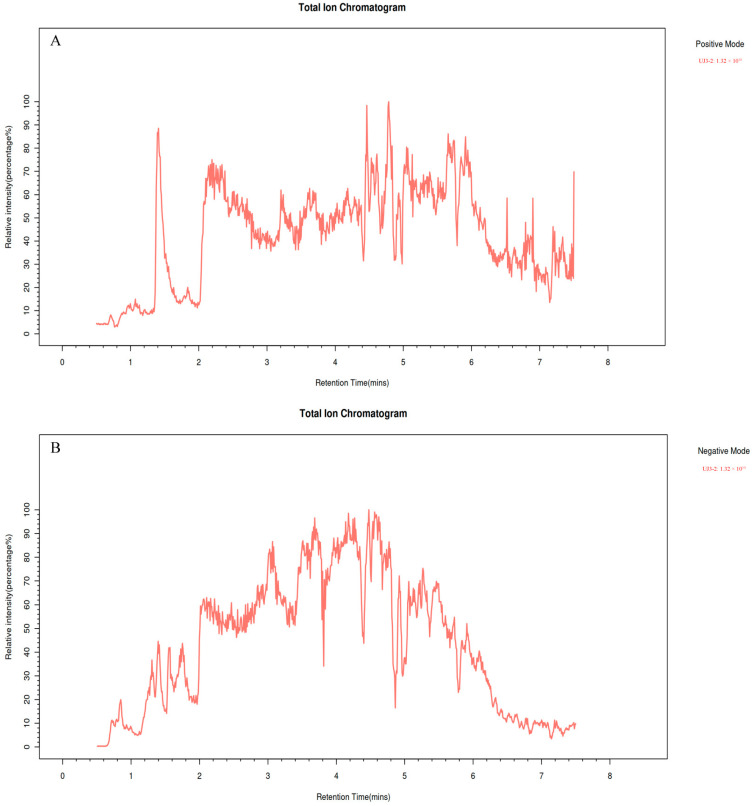
Total ion chromatogram, TIC. (**A**) Positive mode; (**B**) Negative mode.

UPLC-MS/MS was used for identification of the fermentation product of UJ3-2 (Figure 2). The identified compounds mainly included low-molecular-weight phenolic compounds, alkaloids, flavonoids, organic acids, amino acids, sugars, lipids, sugar alcohols, etc. Among them, 6-oxocineole (17.92%), (s)-2-acetolactate (9.91%), 3-methyl-cis,cis-muconate (4.36%), and 8-oxogeranial (3.17%) had the highest concentrations (Table 1). Compounds such as solasodine, alpha-curcumene, bornyl isovalerate, 4-hydroxybenzaldehyde, and naringenin exhibit antibacterial activity, while aesculetin exhibits anti-inflammatory and antitumor activities. Additionally, 8-oxogeranial serves as a substrate for the synthesis of the basic chemical structure (iridoid ring scaffold) of iridoid compounds.

### 2.3. MIC and MBC of Fermentation Product of UJ3-2 against S. aureus

As can be seen from Table 2, for *S.aureus*, when the concentration of UJ3-2 fermentation product is 1.563 mg/mL, the liquid medium is cloudy, indicating that there is significant growth of *S. aureus*. However, when the mass concentration is 3.125 mg/mL, the culture medium is clear, indicating no significant growth of the strain. Therefore, the MIC of the UJ3-2 fermentation product against this indicator bacterium can be determined to be 3.125 mg/mL. According to Table 2, the MBC of the UJ3-2 fermentation product against this indicator bacterium can also be determined to be 3.125 mg/mL.

### 2.4. Effects of UJ3-2 Fermentation Products on Growth of S. aureus

**Figure 3:**
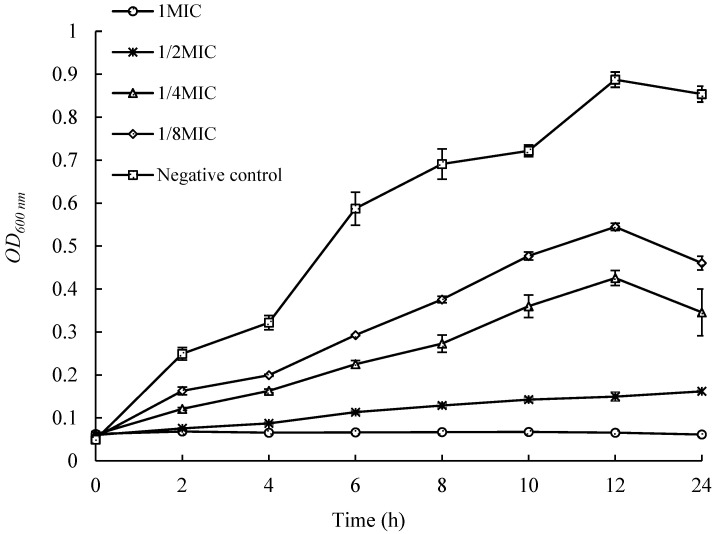
Effects of UJ3-2 fermentation products on growth of *S. aureus*.

As shown in Figure 3, the control group strain grew rapidly within 12 h, reaching an OD_600nm_ of 0.887 at 12 h. The growth of strains with the addition of different concentrations of extracts was significantly inhibited, and their OD values, at the same time, were significantly lower than those of the control group. When the concentration of the added extract reached 1 MIC, the growth of the strain was almost completely inhibited, with no significant change in OD_600nm_ within 24 h. This indicates that when the concentration of the extract from UJ3-2 metabolites reaches 1 MIC, it can effectively inhibit the growth and reproduction of *S. aureus* strains in liquid culture.

### 2.5. Effects of UJ3-2 Fermentation Products on Growth of S. aureus Biofilm

**Figure 4:**
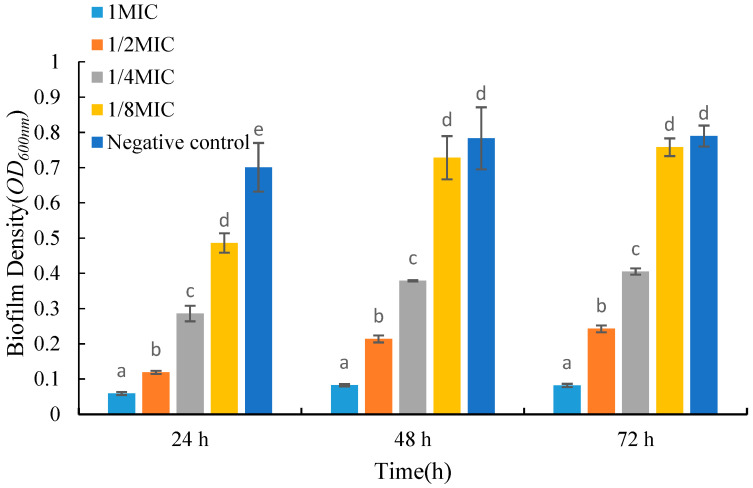
Effects of UJ3-2 fermentation products on growth of *S. aureus* biofilm. At the same time point, different letters represented significant difference (*p* < 0.05).

As shown in Figure 4, the biomass of biofilms decreased significantly after adding the fermentation product to the medium. For biofilms grown in the same period, the biomass gradually decreased with an increase in the concentration of the fermentation product. Compared with the control group, the absorbance values of 24 h old biofilms treated with low and high concentrations of fermentation products decreased by 30.7% and 91.5%, respectively. After 72 h of growth, the absorbance of biofilms treated with low and high concentrations of fermentation products decreased by 4.01% and 89.6%, respectively. From Figure 4, it can be seen that when treated with the same concentration of fermentation product, the biomass of 48 h old biofilms is larger than that of 24 h old biofilms, but there is no significant trend in the biomass of 72 h old biofilms compared to 48 h old biofilms.

### 2.6. Effects of UJ3-2 Fermentation Products on Extracellular Nucleic Acid and Protein of S. aureus

**Figure 5:**
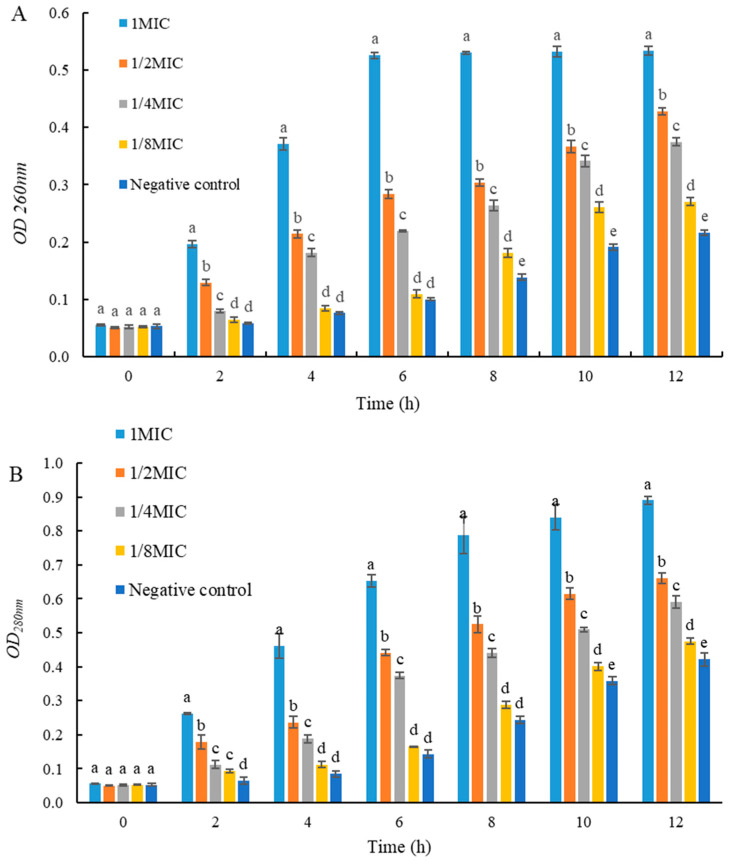
Effects of UJ3-2 fermentation products on extracellular nucleic acid and protein of *S. aureus.* (**A**) OD_260nm_; (**B**) OD_280nm_. At the same time point, different letters represented significant difference (*p* < 0.05).

As shown in Figure 5, the concentrations of extracellular nucleic acids and proteins in *S. aureus* in the 1 MIC and 2 MIC groups were significantly higher than those in the control group (*p* < 0.05). This result indicates that the fermentation product of endophytic fungus UJ3-2 can disrupt the cell membrane structure of *S. aureus*, causing the leakage of intracellular nucleic acids and proteins from the bacteria.

### 2.7. Effects of UJ3-2 Fermentation Products on Cell Membrane Integrity of S. aureus

**Figure 6:**
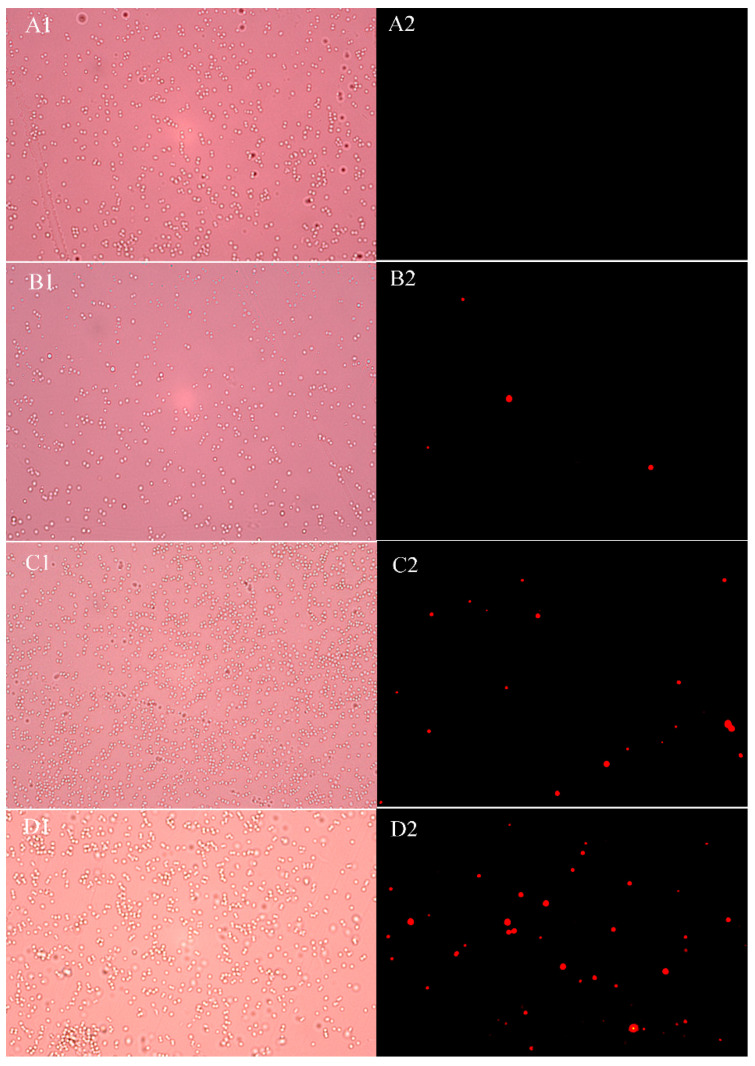
Effects of different concentrations of UJ3-2 fermentation product on cell membrane integrity of *S. aureus* (fluorescence microscope). Note: (**A**) negative control group ((**A1**) bright field, (**A2**) dark field); (**B**) 1/4 MIC group ((**B1**) bright field, (**B2**) dark field); (**C**) 1/2 MIC group ((**C1**) bright field, (**C2**) dark field); (**D**) 1 MIC group ((**D1**) bright field, (**D2**) dark field).

Propidium iodide (PI), as a membrane-impermeable fluorescent dye, possesses a unique mechanism of action [15]. When the integrity of the cell membrane is compromised, it can penetrate the membrane, bind to genetic material, and emit red fluorescence. Therefore, the integrity of the cell membrane can be reflected by detecting PI fluorescence through fluorescence microscopy. As shown in Figure 6, after PI staining, the control group exhibited virtually no observable fluorescence, while the *S. aureus* group treated with 0.5 MIC metabolites for 4 h emitted sporadic red fluorescence. With an increase in extract concentration, the red fluorescence gradually intensified. When the mass concentration reached 2 MIC, a large amount of red fluorescence was observed, indicating that after treatment with the extract, the cell membrane of *S. aureus* was disrupted, allowing a significant amount of PI to enter the cells and bind to genetic material, resulting in the emission of red fluorescence.

### 2.8. Effect of Fermentation Products on S. aureus Was Observed by Scanning Electron Microscope

**Figure 7:**
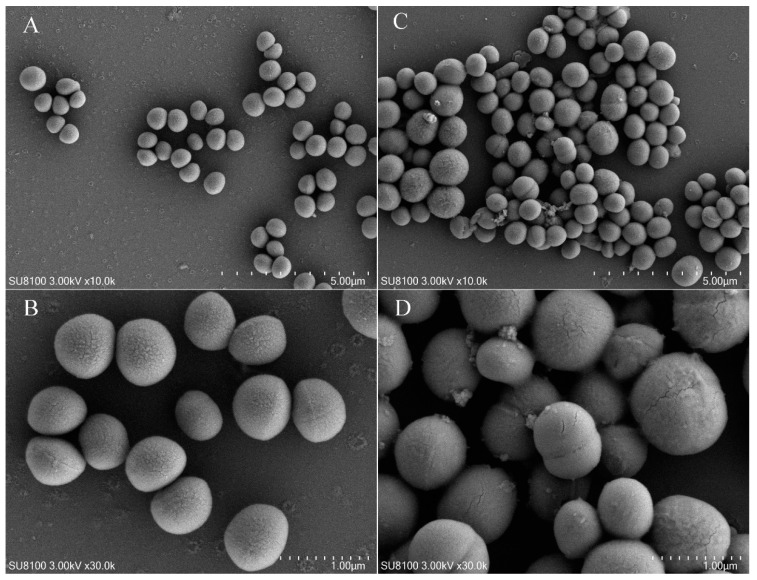
Effect of 1/2 MIC of UJ3-2 fermentation product on morphology of *S. aureus* cells. Note: (**A**,**B**) normal controls; (**C**,**D**) processing group.

Observations from scanning electron microscopy revealed that compared to the control group (Figure 7A,B), *S. aureus* treated with the fermentation product of UJ3-2 exhibited bacterial morphological deformations, including blurred edges and rough surfaces. This indicates that the fermentation product of UJ3-2 can affect the overall morphology of *S. aureus* and alter its bacterial surface structure.

## 3. Discussion

The secondary metabolites of microorganisms, as natural antibacterial substances, are important sources for the development and creation of new drugs [16]. Endophytic fungi in plants are highly diverse groups in microbial classification [17]. These fungi can synthesize medicinal substances. Therefore, they may be excellent sources of new antibacterial compounds for the treatment of human and animal pathogen infections [18]. However, the study of these microbial resources is boundless and requires the extensive attention of microbiologists and medicinal chemists. In this study, an endophytic fungus, UJ3-2, was isolated from fresh plants of *Urtica fissa* (*U. fissa*). By means of ITS sequencing and alignment, it was identified as *Xylaria grammica* fungus. *U. fissa* is commonly used to treat rheumatism, arthritis, and urticaria. Many chemical compounds with anti-inflammatory properties have recently been identified [19,20,21,22,23]. Fungi belonging to the genus *Xylaria* are concentrated in temperate, subtropical, and tropical regions, and their main chemical components are secondary metabolites such as terpenes, alkaloids, sterols, and polyketides, which possess a variety of pharmacological activities, including antioxidation, antibiosis, antitumor activity, etc. [24]. *Xylaria* that symbiotizes with plants can also produce the same or similar physiologically active substances as its host plants [25]. An endolichenic fungus, *Xylaria grammica* strain EL000614, exhibited potent nematicidal activity against the plant-pathogenic nematode Meloidogyne incognita through the production of grammicin [26,27].

The strain UJ3-2 obtained in this study exhibited significant inhibitory effects on *S. aureus*. The MIC of the extract from its fermentation broth against *S. aureus* was 3.125 mg/mL. When the concentration of the extract was 1/8 MIC, it significantly inhibited the growth of *S. aureus*, with a notably lower OD_600_ value compared to the control group at the same time point. At a concentration of 1 MIC, it completely inhibited the growth of *S. aureus*. When the extract concentration was 1/4 MIC, it significantly inhibited the biofilm formation of *S. aureus*. To investigate the effect of the UJ3-2 fermentation broth on the cell membrane integrity of *S. aureus*, the bacteria were stained with PI and observed under a fluorescence microscope. PI cannot penetrate intact cell membranes, but when the cell membrane is damaged, PI can enter the cell, stain the DNA, and emit red fluorescence [28]. As the concentration of the fermentation broth increased, the damage to the cell membrane of *S. aureus* became more severe. When the concentration of the fermentation broth reached 1 MIC, the cell membrane of *S. aureus* was severely damaged, resulting in a significant number of bacterial deaths. Additionally, scanning electron microscopy revealed that *S. aureus* treated with the fermentation product of UJ3-2 exhibited morphological deformations, including blurred edges and rough surfaces, indicating that the fermentation product of UJ3-2 can affect the overall morphology and alter the bacterial surface structure of *S. aureus*. Microbial biofilm is an aggregate of microbial cells embedded in the extracellular polymeric substance (EPS) matrix produced by themselves. Biofilm is resistant to extreme environments and can protect microorganisms from ultraviolet (UV) radiation, extreme temperatures, extreme pH values, high salinity, high pressure, malnutrition, antibiotics, etc., by acting as an “aprotective suit” [29]. Therefore, it is speculated that the antibacterial mechanism of the UJ3-2 fermentation broth against S. aureus may be attributed to the increased concentration of the fermentation broth, which leads to enhanced permeability of the cell membrane of *S. aureus*, damaging the integrity of the cell membrane. This results in the influx of a large number of exogenous substances and the leakage of cytoplasm, causing intracellular osmotic pressure disorders that prevent normal growth and reproduction of the bacteria.

UPLC-MS was used to analyze and identify the components of the UJ3-2 fermentation broth extract. The identification results showed that the UJ3-2 fermentation broth extract mainly contained compounds such as organic acids, flavonoids, phenols, coumarins, and alkaloids. The content of 6-oxocineole (17.92%), (S)-2-acetolactate (9.91%), 3-methyl-cis,cis-muconate (4.36%), and 8-oxogeranial (3.17%) was the highest. 6-oxocineole is a cineole. Cineole, a terpene oxide inherent in a myriad of plant essential oils, boasts notable anti-inflammatory, analgesic, and antioxidant properties [30,31]. 8-Oxogeranial serves as a precursor in the biosynthesis of iridoids. Iridoid synthases (IRIS), members of the PRISE subclass within the SDR protein family, orchestrate the pivotal step in iridoid formation: the cyclization of 8-oxogeranial into nepetalactol, the ubiquitous biosynthetic precursor for all iridoids [32]. The fundamental chemical architecture of iridoids in plants, namely the iridoid ring scaffold, is biologically synthesized within the plant by the enzyme iridoid synthase, employing 8-oxogeranial as its primary substrate [33]. Iridoids exhibit a promising capacity to modulate numerous biological processes in a wide array of diseases [34]. Among them, compounds such as solasodine, alpha-Curcumene, Bornyl isovalerate, 4-hydroxybenzaldehyde, and naringenin exhibit antibacterial activity. Da Silva has shown that solasodine may be acting on the mechanism of action of the antibiotic, as it has shown a potentiating effect when associated with antibiotics, inducing a reduction in the MIC against Gram-positive and Gram-negative bacteria [35]. Solasodine can be used as a potent anti-mycobacterial agent for combating *Paratuberculosis* [36]. Zhang et al. have shown that ginger essential oil can disrupt the cell membrane and biofilm structure of *Staphylococcus*, demonstrating its potential as a natural food preservative. Among its components, alpha-curcumene is the main active compound of ginger essential oil [37]. 4-hydroxybenzaldehyde sensitizes *Acinetobacter baumannii* to amphenicols [38]. Naringenin has antidiabetic, anticancer, antimicrobial, antiobesity, gastroprotective, immunomodulator, cardioprotective, nephroprotective, and neuroprotective properties [39,40]. This indicates that the components of the UJ3-2 fermentation extract are complex and abundant, exerting an antibacterial effect through multiple components.

## 4. Materials and Methods

### 4.1. Materials

The endophytic fungus UJ3-2 of *Urtica fissa* was previously isolated by our laboratory; the strain of *S. aureus* (CMCC(B)26003) was purchased from Shanghai Luwei Technology Co., Ltd. (Shanghai, China); Casein Hydrolysate (MH) broth medium and LB agar medium were provided by Qingdao Haibo Biotechnology Co., Ltd. (Qingdao, China); propidium iodide (PI) was obtained from Solarbio (Beijing, China); and anhydrous ethanol, ethyl acetate, ether, and n-butanol (analytical pure) were obtained from Aladdin (Shanghai, China).

### 4.2. Methods

#### 4.2.1. Identification of Endophytic Fungi

The identification of isolated endophytic fungi was carried out using the ITS (Internal Transcribed Spacer) sequence of *Urtica fissa*. Universal fungal primers were employed to amplify the ITS region [41]: ITS1: 5′-TCCGTAGGTGAACCTGCGG-3′, and ITS4: 5′-TCCTCCGCTTATTGATATGC-3′. The resulting ITS sequences were then submitted to the GeneBank database, and BLAST on the NCBI website was used to search for the DNA sequence with the highest similarity. Together with the sequencing results, the software Mega11 version 11.0.11 was utilized to construct an evolutionary tree using the neighbor-joining (NJ) method, in order to identify the generic classification of each endophytic fungal strain. 

#### 4.2.2. Preparation of UJ3-2 Fermentation Products

The fungus was inoculated in a conical flask containing Potato Dextrose Broth and cultured in a constant-temperature shaker for 15 days (28 °C, 120 r/min) to obtain a fermented broth for later use. The fermented broth was centrifuged (4 °C, 8000 r/min) for 10 min, and the supernatant was retained. On a sterile working bench, the supernatant was filtered through a 0.22 μm filter membrane to obtain a sterile fermented broth. The sterile fermented broth was then extracted with an equal volume of ethyl acetate for 30 min each time, and this was repeated three times. The combined extracts were concentrated using a rotary evaporator under reduced pressure and then dried in a vacuum oven to obtain the fermented metabolites of UJ3-2.

#### 4.2.3. Ultra-High-Performance Liquid Chromatography–Mass Spectrometry (UPLC-MS)

**Chromatographic Separation:** A Thermo Vanquish (Thermo Fisher Scientific, Waltham, MA, USA) ultra-high-performance liquid chromatography (UHPLC) system was employed, utilizing an ACQUITY UPLC^®^ HSS T3 (2.1 × 100 mm, 1.8 µm) column (Waters, Milford, MA, USA) with a flow rate of 0.3 mL/min and a column temperature set at 40 °C. A sample volume of 2 μL was injected. For the positive ion mode, the mobile phases consisted of 0.1% formic acid in acetonitrile (B2) and 0.1% formic acid in water (A2), with the following gradient elution program: 0–1 min, 10% B2; 1–5 min, 10%–98% B2; 5–6.5 min, 98% B2; 6.5–6.6 min, 98%–10% B2; 6.6–8 min, 10% B2. For the negative ion mode, the mobile phases were acetonitrile (B3) and 5 mM ammonium formate in water (A3), with the following gradient elution program: 0–1 min, 10% B3; 1–5 min, 10%–98% B3; 5–6.5 min, 98% B3; 6.5–6.6 min, 98%–10% B3; 6.6–8 min, 10% B3.

**Mass Spectrometry Acquisition:** A Thermo Q Exactive Focus mass spectrometer (Thermo Fisher Scientific, USA), equipped with an Electrospray Ionization (ESI) source, was used to acquire data in both positive and negative ion modes. The spray voltages were set at 3.50 kV for positive ions and −2.50 kV for negative ions. The sheath gas flow rate was 40 arb, and the auxiliary gas flow rate was 10 arb. The capillary temperature was maintained at 325 °C. A first-order full scan was performed at a resolution of 70,000, with an ion scan range of *m*/*z* 100–1000. Higher-energy collisional dissociation (HCD) was employed for second-order fragmentation, with a collision energy of 30 eV and a second-order resolution of 17,500. The top 3 most abundant ions were selected for fragmentation, and dynamic exclusion was applied to remove unnecessary MS/MS information [42].

#### 4.2.4. Measurement of Minimum Inhibitory Concentration (MIC) and Minimum Bactericidal Concentration (MBC)

Using the two-fold dilution method, 100 μL of culture medium was pre-added to wells 2 to 8 in a 96-well plate. A total of 200 μL of extract solution was added to well 1, and 100 μL of the sample solution was withdrawn and added to well 2; this pattern was followed for serial two-fold dilutions until well 8. To wells 1 to 8, 10 μL of 1 × 10^5^ CFU/mL bacterial suspension was added sequentially. Well 9 contained an equal volume of culture medium, bacterial suspension, and normal saline, with the latter serving as the control group. Well 10 contained only culture medium, serving as the blank control. After completing the sample addition, the plates were incubated at 37 °C for 24 h. The MIC was determined by visually inspecting the wells for turbidity. For each group, this process was repeated three times. Based on the MIC test results, 10 μL of solution from each well was plated, and the plates were incubated at 37 °C for 24 h to observe colony growth on the agar plates. The MBC of the fermentation extract was defined as the lowest drug concentration at which no colonies grew [43].

#### 4.2.5. Determination of Growth Curve of *S. aureus* by Fermentation Extract of UJ3-2

We added 100 μL of bacterial suspension (1 − 2 × 10^7^ CFU/mL) to a 96-well cell culture plate, and then added 100 μL of extract at concentrations of 2 MIC, 1.0 MIC, and 0.5 MIC to the respective wells, resulting in final mass concentrations of 0.25 MIC, 0.5 MIC, and 1.0 MIC of the fermentation extract in the wells. We used culture medium instead of extract as a control, with 4 wells for each concentration. We incubated the 96-well cell culture plate at 35 °C for 16 h, measuring OD_600nm_ every 2 h. We plotted a growth curve with time as the horizontal axis and optical density values as the vertical axis [44].

#### 4.2.6. Determination of the Biofilm of *S. aureus* by Fermentation Extract of UJ3-2

To measure the inhibitory effect of UJ3-2 fermentation extract on *S. aureus* biofilm, 2.97 mL of TSB (Tryptic Soy Broth) medium was added to surface-treated 12-well plates and inoculated with 30 μL of *S. aureus* suspension at 10^9^ CFU/mL. Fermentation products were then added to achieve final concentrations of 0.5, 1, and 5 mg/mL, respectively. The wells without fermentation products were considered the control group. The 12-well plates were incubated at 37 °C for 24, 48, and 72 h. After incubation, excess bacterial suspension was aspirated, and the wells were washed with PBS to remove free bacteria. Subsequently, 50 μL of methanol was added to each well to fix the biofilm for 30 min. Following fixation, the methanol was discarded, and the wells were rinsed with PBS. Then, 500 μL of 0.5% (*w*/*v*) crystal violet solution was added to stain the biofilm for 15 min. After staining, excess crystal violet solution was discarded, and the wells were washed with PBS. The plates were dried, and 300 μL of 70% ethanol was added. Once the crystal violet in the wells was fully dissolved, the absorbance at OD_600nm_ was measured [45].

#### 4.2.7. Detection of Extracellular Nucleic Acid and Protein of *S. aureus* by Fermentation Extract of UJ3-2

The bacterial solution cultured to the logarithmic growth phase was washed with sterile PBS, resuspended, and divided into four tubes. UJ3-2 fermentation extract was added to the resuspended solution at final concentrations of 1, 1/2, 1/4, and 1/8 MIC, respectively, and sterile PBS was used to set up a negative control. The samples were incubated in a 37 °C water bath for 12 h. At 0, 2, 4, 6, 8, 10, and 12 h, 200 μL of the samples was taken and centrifuged at 4000 rpm for 5 min. The supernatants were collected to measure the OD_260_ and OD_280_ values [46].

#### 4.2.8. Effects of UJ3-2 Fermentation Extract on Membrane Integrity of *S. aureus*

Using the propidium iodide (PI) staining method, 500 μL of bacterial suspension was added to 2 mL centrifuge tubes. Then, 500 μL of the extract solutions at concentrations of 1 MIC, 2 MIC, and 4 MIC was added to the centrifuge tubes, resulting in final extract concentrations of 0.5 MIC, 1 MIC, and 2 MIC, respectively. Sterile deionized water was used instead of the extract as a control, with 3 tubes for each concentration. The samples were incubated at 28 °C for 4 h. The cultured bacterial suspensions were centrifuged at 5000 rpm for 10 min at 4 °C, and the supernatants were discarded. The obtained bacterial cells were resuspended and washed twice with PBS, followed by centrifugation to discard the supernatant again. The bacterial cells were resuspended in PBS, and PI dye (final concentration of 10 μg/mL) was added. The samples were then incubated in the dark at 4 °C for 30 min. Afterward, the bacterial suspensions were resuspended, washed twice with PBS, centrifuged to discard the supernatant, and resuspended in PBS. Finally, 10 μL of bacterial suspension was taken and observed under a fluorescence microscope with an excitation wavelength of 535 nm and an emission wavelength of 615 nm [47].

#### 4.2.9. Cell Membrane Integrity of *S. aureus*

The bacterial suspension cultured to the logarithmic growth phase was washed and resuspended using sterile PBS. Fungal fermentation products were added to achieve a final concentration of 1/2 MIC, while sterile PBS was used to set up a negative control. The samples were incubated in a 37 °C water bath for 4 h, and then centrifuged at 4000 rpm for 5 min. The supernatants were discarded, and the bacterial cells were washed twice with sterile PBS. The bacterial cells were resuspended in electron microscopy fixative, fixed at room temperature for 2 h, and then stored at 4 °C. After being left overnight, the bacteria were washed with PBS 3 times and fixed in 1% osmic acid for 30 min, and then gradient elution with different concentrations of ethanol. Finally, the samples were added to a special slide and attached to a field emission scanning electron microscope (SEM) (Hitachi, Tokyo, Japan) stage. After drying for 8 h with a critical point dryer, the samples were gold-plated. The morphology of bacterial cells was observed with the SEM [48].

### 4.3. Statistical Analyses

Microsoft Office 2016 software was used for creating figures, and SPSS Statistics 20.0 (IBM Corp., Armonk, NY, USA) was used for conducting an ANOVA analysis and *t*-test on the data. The experiment was repeated three times, and the average of the measured results was taken. The data are presented as means ± SD.

## 5. Conclusions

The present study underscores the remarkable antibacterial prowess of the metabolites sourced from *Xylaria grammica* UJ3-2 against *S. aureus*, a prevalent cause of infections worldwide. By effectively targeting and inhibiting the formation of robust *S. aureus* biofilms, as well as disrupting the intricate architecture and stability of the bacterial cell wall, these metabolites elicit the release of crucial intracellular constituents, such as nucleic acids and proteins, to varying degrees. This leakage not only disrupts the normal metabolic activities but also significantly impedes the growth and propagation of the bacteria. Thus, this study delves into the initial stages of elucidating the mechanistic pathways underpinning the antibacterial effects of *Xylaria grammica* UJ3-2 on *S. aureus*, offering valuable insights and serving as a cornerstone for future endeavors aimed at harnessing natural compounds as innovative antimicrobial agents to counter the rising tide of antibiotic resistance among bacterial pathogens.

## Figures and Tables

**Table 1:** Main components of fermentation product of UJ3-2.

Compound	Formula	Mz	Rt (S)	Relative Content (%)
6-oxocineole	C_10_H_16_O_2_	213.1127	275.7	17.92%
(S)-2-acetolactate	C_5_H_8_O_4_	171.1375	297.6	9.91%
3-methyl-cis,cis-muconate	C_7_H_8_O_4_	137.0228	161.6	4.36%
8-oxogeranial	C_10_H_14_O_2_	211.095	271.4	3.17%
7-hydroxy-4-isopropenyl-7-methyloxepan-2-one	C_10_H_16_O_3_	185.117	218	3.11%
(S)-4,5-dihydroxypentane-2,3-dione	C_5_H_8_O_4_	113.023	56.2	2.58%
4-hydroxybenzaldehyde	C_7_H_6_O_2_	121.0273	255.3	2.26%
Anandamide	C_22_H_37_NO_2_	304.2991	341.4	2.19%
2-oxohept-3-enedioate	C_7_H_8_O_5_	153.0198	83.7	1.74%
3,5-dihydroxybenzoic acid	C_7_H_6_O_4_	307.0443	136.2	1.65%
Coniferaldehyde	C_10_H_10_O_3_	177.0531	233.9	1.52%
L-dopa	C_9_H_11_NO_4_	179.0351	271.4	1.26%
Methyl gibberellin A9	C_20_H_26_O_4_	311.1681	324.1	1.21%
4-hydroxy-5-methyl-2-methylene-3(2*H*)-furanone	C_6_H_6_O_3_	125.0229	156.1	1.10%
p-hydroxyphenylacetic acid	C_8_H_8_O_3_	135.044	184.8	1.08%
Gentisic acid	C_7_H_6_O_4_	307.0461	131.2	1.07%
7-chlorotryptophan	C_11_H_11_CIN_2_O_2_	237.0399	211.2	1.01%
Aesculetin	C_9_H_6_O_4_	355.0451	202.8	0.94%
Tridecanoic acid	C_13_H_26_O_2_	427.2343	284.2	0.93%
Norfuraneol	C_5_H_6_O_3_	227.0496	88.3	0.89%
4-acetoxy-2-hexyltetrahydrofuran	C_12_H_22_O_3_	237.1117	222.5	0.84%
Solasodine	C_27_H_43_NO_2_	396.3254	270.8	0.76%
Bombykol	C_16_H_30_O	256.2628	433	0.68%
4-hydroxy-3-methylbenzoic acid	C_8_H_8_O_3_	151.0383	195.4	0.63%
Bornyl isovalerate	C_15_H_26_O_2_	221.1897	338	0.59%
latiluciferin	C_15_H_24_O_2_	237.1844	325.2	0.58%
Pyruvic acid	C_3_H_4_O_3_	133.0138	154.9	0.54%
3-hydroxymandelic acid	C_8_H_8_O_4_	167.034	127.3	0.52%
Alpha-curcumene	C_15_H_22_	203.179	268.9	0.51%
Chrysanthemic acid	C_10_H_16_O_2_	167.0533	300	0.49%
8-epiiridodial	C_10_H_16_O_2_	169.1209	289.1	0.49%
3,4-dimethoxybenzaldehyde	C_9_H_10_O_3_	165.0537	172.8	0.47%
Erythronic acid	C_4_H_8_O_5_	117.018	370.3	0.46%
2,5-furandicarbaldehyde	C_6_H_4_O_3_	142.0496	101.7	0.45%
FA 6_2;O	C_6_H_8_O_3_	109.0279	202.7	0.44%
FA 10_2;O	C_10_H_16_O_3_	229.1078	239.3	0.43%
Bisphenol A	C_15_H_16_O_2_	212.0947	126.2	0.39%
Nepetalactone trans-cis-form	C_10_H_14_O_2_	184.1692	96.5	0.37%
10-OPDA	C_18_H_28_O_3_	337.2035	350.5	0.37%
Naringenin	C_15_H_12_O_5_	271.0604	271.6	0.37%
1,2-dimethyl-4-(6-methyl-4-heptenyl)-1,3-cyclohexadiene	C_16_H_26_	219.1732	382	0.36%
Total				70.66%

Mz = mass-to-charge ratio; Rt = retention time.

**Table 2:** MIC and MBC of UJ3-2 fermentation product against *S. aureus*.

Project	Concentration (mg/mL)								Control	MIC (mg/mL)	MBC (mg/mL)
	50.00	25.00	12.50	6.250	3.125	1.563	0.781	0.391			
Growing states	−	−	−	−	−	+	+	+	+	3.125	
	−	−	−	−	−	+	+	+	+		3.125

Note: “−” indicates antibacterial activity, and “+” indicates bacterial growth.

## Data Availability

The original contributions presented in the study are included in the article.
